# COVID-19 and Interstitial Lung Disease

**DOI:** 10.3390/medicina62010022

**Published:** 2025-12-23

**Authors:** Roberto G. Carbone, Sharada Nagoti, Assaf Monselise, Keith M. Wille, Francesco Puppo, Pallav L. Shah

**Affiliations:** 1 Department of Internal Medicine, University of Genoa, 16132 Genoa, Italy; puppof@unige.it; 2 Department of Pathology, Wadia Hospital for Children, Mumbai 400014, Maharashtra, India; drsharada.lungpath@gmail.com; 3 Clalit Health Service, Tel Aviv 6209804, Israel; a.mons@yahoo.com; 4 Department of Medicine, University of Alabama at Birmingham, Birmingham, AL 35294, USA; kwille@uabmc.edu; 5 National Institute for Health Research Unit, Royal Brompton and Harefield NHS Foundation Trust, London SW3 6PY, UK; pallav.shah@imperial.ac.uk

**Keywords:** SARS-CoV-2, COVID, post-COVID ILD, interstitial lung disease, ILD, idiopathic pulmonary fibrosis, lung cryobiopsy, high resolution chest computed tomography, antifibrotic drugs

## Abstract

*Background and Objectives*: COVID-19 is an infection caused by the SARS-CoV-2 coronavirus that may develop several complications. Interstitial lung disease (ILD) is the major long-term complication of COVID-19 disease leading to progressive lung fibrosis and reduced respiratory function. The aim of this narrative review is to provide an updated overview of post-COVID-19 ILD by examining research publications and clinical guidelines selected from PubMed, Web of Science, and major respiratory medicine journals from 2020 to 2025. *Methods*: ILDs are diagnosed by medical history, physiological examination, pulmonary function tests, and chest X-ray or high-resolution computed tomography (HRCT) scan. Lung biopsy, especially cryobiopsy or video-assisted thoracoscopic (VATS) biopsy, can be performed to define histological patterns and confirm the diagnosis. *Results*: Post-COVID-19 ILD is a chronic condition characterized by long-term respiratory symptoms, radiological findings, and reduced lung function. Fibrotic injury is a consequence of the initial infection and could be influenced by persistent inflammation and dysregulated tissue repair. Risk factors include severe acute illness, advanced age, male sex, and smoking. Clinical course and prognosis of post-COVID-19 ILD is uncertain, as most patients experience gradual improvement or stability, whereas others develop progressive lung function decline. Treatment of post-COVID-19 ILD is not presently defined by guidelines but comprises corticosteroids, antifibrotics (including new drugs such as nerandomilast), supportive oxygen, pulmonary physiotherapy rehabilitation, smoking cessation, and vaccination. *Conclusions:* ILD represents a significant long-term complication of COVID-19 infection. Further investigations are required to better understand its pathophysiology and clinical management. As research progresses, more effective diagnostic and therapeutic strategies are expected to emerge.

## 1. Introduction

**Figure 1:**
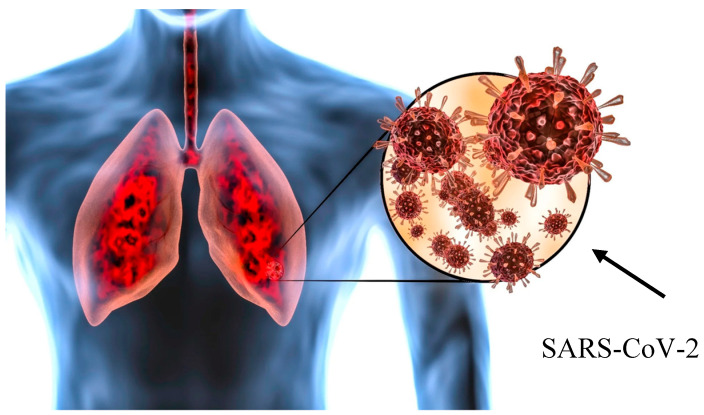
Picture depicting lung involvement in COVID-19 infection leading to interstitial lung disease.

COVID-19 disease is an infection caused by the SARS-CoV-2 coronavirus that may develop subsequent complications involving different organ systems after recovery. These complications include myocarditis, myositis, and brain, kidney, and liver disorders. Pulmonary fibrosis is the major long-term complication of COVID-19 disease (Figure 1).

Pulmonary fibrosis is a progressive and fatal chronic disease that causes lung scarring (fibrosis). It is characterized by interstitial collagen deposition with varying degrees of alveolar bronchiolization [1]. The prevalence of post-COVID-19 interstitial lung disease (ILD) is 44.9%, and characteristic features include airway remodeling and lung function decline [2].

## 2. Methodology

This narrative review synthesizes the current knowledge on post-COVID-19 ILD by examining peer-reviewed publications, clinical guidelines, and recent research studies. The literature sources are selected from PubMed, Web of Science, and major respiratory medicine journals, focusing on publications from 2020 to 2025. Search terms included “COVID-19,” “SARS-CoV-2,” “interstitial lung disease,” “pulmonary fibrosis,” “post-COVID complications,” and related terms. We prioritize high-quality studies including randomized controlled trials, systematic reviews, cohort studies, and expert consensus statements. This review integrates clinical, radiological, histopathological, and therapeutic perspectives to provide a comprehensive overview of post-COVID-19 ILD within the broader context of fibrosing lung disorders.

## 3. Pulmonary Fibrosis Mechanisms

Infection with SARS-CoV-2 triggers acute lung injury accompanied by intense alveolar inflammation. Notably, a significant proportion of COVID-19 patients develop Acute Respiratory Distress Syndrome (ARDS) leading to extensive lung injury. The lung responds to inflammatory damage by producing scar tissue (fibrosis), which replaces healthy lung tissue. The alveolar inflammatory response to SARS-CoV-2 induces excess matrix production, causing post-COVID-19 pulmonary fibrosis. However, the exact pathophysiological mechanisms of post-COVID-19 pulmonary fibrosis remain unclear, and the cause may be multi-factorial. The virus may damage the lungs directly or produce an inflammatory process leading to fibrosis. Plasma proteomics analysis identified 35 proteins closely involved in pathways associated with cell proliferation, tissue remodeling, inflammation, immune response, angiogenesis, and fibrosis. Further, García-Hidalgo et al. found that genes encoding the proteomic profile were enriched in lung epithelial, endothelial, and immune cells [3]. Proteins involved in lung fibrosis are not related to a single pathogenetic process but rather to a complex interplay between inflammation and remodeling. A dysregulated phenomenon of repair mechanisms, including fibroblasts and myofibroblasts, lead to excessive secretion of the extracellular matrix [4]. This fibrotic process occurs particularly in alveolar lung regions, leading to increased airway wall thickness and loss of alveolar tissue, resulting in ventilation–perfusion disorders and hypoxemia.

## 4. Post-COVID-19 Interstitial Lung Disease

The most common post-COVID-19 respiratory ailment is ILD, a heterogeneous group of respiratory disorders characterized by fibrosis and inflammation of the lung interstitium. Of the ILDs, the subset with the highest prevalence and incidence reported among all world registries is idiopathic pulmonary fibrosis (IPF) [5].

IPF is a disease of unknown origin with histological features corresponding to usual interstitial pneumonia (UIP). It is the most common subtype of idiopathic interstitial pneumonia (IIP) and is associated with the highest mortality rate. Current therapies have improved IPF outcomes [6,7,8,9]. However, IPF patients in the UK continue to face a poor prognosis, with a median survival of 3–5 years following diagnosis and an average loss of 7 years in life expectancy [7]. Survival of patients with IPF-UIP is lower than that for patients with nonspecific interstitial pneumonia (NSIP) [10]. Mortality studies in patients with IPF-UIP and NSIP have addressed a variety of possible prognostic factors. However, these studies were limited by their retrospective nature, shorter median observation time, and limited number of variables included [11].

In this context, the majority of IPF patients did not improve with conventional therapy (corticosteroids and cytotoxic agents) and suffered from serious side effects. Clinical trials proposed new treatment strategies for IPF, such as the antifibrotic drugs pirfenidone and nintedanib [8,9]. Lung transplantation has proven to be the only intervention capable of increasing life expectancy in IPF patients [12]. Given the variable course of IPF and lack of validated prognostic measures, the optimal timing for transplantation is not yet well-defined.

Validation of emerging prognostic factors may help determine the right timing to initiate antifibrotic therapy and lung transplantation, as well as to better stratify patients in future clinical trials. However, a comprehensive scoring system that includes all recognized IPF variables is currently lacking. In fact, current scores compare IPF severity with one or two variables, such as pulmonary function tests, HRCT scans, or exacerbations. Additionally, pulmonary hypertension is underestimated or not considered in IPF data analyses. Furthermore, a standard definition of disease progression and rate of decline has not been formally accepted. Therefore, it is difficult to predict the disease course and establish appropriate treatment modalities.

Numerous studies have attempted to identify predictors of outcome in IPF patients [13,14,15]. Risk factor modeling and scoring systems will allow better prediction of disease progression and appropriate timing for antifibrotic therapy or lung transplantation.

## 5. ILD Epigenetics and COVID-19

Current epigenetic studies investigate heritable changes in gene expression that occur without changes in DNA sequence. Several epigenetic mechanisms, including DNA methylation, histone modifications, and microRNAs, can change the genome function under exogenous influence. Recent research discusses and defines topics that must be addressed to investigate the role of epigenetics in human health and disease [16,17,18].

In this context, some of the major unresolved questions include: (i) what are the roles of noncoding RNAs as epigenetic mechanisms following vascular lung injury; (ii) what are the epigenetic modifications in parenchymal lung injury, pulmonary fibrosis, pulmonary hypertension, and airways diseases; (iii) what is the evidence for epigenetic involvement after lung and other solid organ transplantation and their association with immune responses; (iv) what are the primary biomarkers of lung injury after transplantation. Of note, epigenetic studies on pulmonary arterial hypertension, chronic obstructive pulmonary disease, interstitial lung disease, and lung cancer can help improve present and future knowledge of COVID-19-related complications.

## 6. ILD Clinical Assessment and Diagnostic Procedures

### 6.1. Clinical Assessment

Symptoms of ILD associated with COVID-19 depend on disease severity. They may include shortness of breath, cough, fatigue, chest pain, and wheezing. ILD and especially IPF are diagnosed by medical history, physiological examination, pulmonary function tests, and imaging such as chest X-ray or high-resolution computed tomography (HRCT) scanning. Lung biopsy can be performed to confirm the diagnosis. Patients at higher risk of developing ILD are older, have severe respiratory failure, are recovering from pneumonia or ARDS, or have a restrictive respiratory pattern with low forced vital capacity (FVC), TLC, and DLCO. Males are at greater risk of ILD development after COVID-19 infection. Patients affected by severe ILD may require non-invasive or mechanical ventilation [19,20].

Importantly, the cardiovascular system plays a critical role in the lung’s response to severe viral injury. Patients with pre-existing cardiovascular disease (CVD) demonstrate worse pulmonary outcomes following COVID-19 infection. The interplay between vascular pathology and parenchymal lung injury may accelerate the progression to ILD. Cardiovascular comorbidities serve as significant biomarkers for an increased risk of severe COVID-19 infection and poor prognosis, underscoring the importance of comprehensive cardiovascular assessment in post-COVID-19 ILD patients [21].

### 6.2. Pulmonary Function Tests

**Figure 2:**
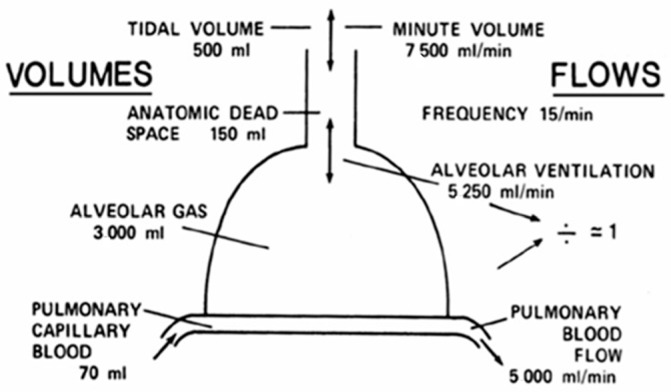
Schematic drawing that shows volumes/flows curve.

Post-COVID-19 pulmonary fibrosis compromises lung tissue with functional abnormalities that reduce lung function, particularly total lung capacity (TLC) and diffusion capacity of the lung for carbon monoxide (DLCO). Therefore, long-term follow up is necessary to evaluate for the progression and extension of irreversible damage. The flow–volume curve is shown in Figure 2.

## 7. Radiological Procedures

### 7.1. Chest Radiography

Chest radiography (CXR) may show signs of inflammation-related lung injury as ground-glass opacities or fibrosis in patients with severe respiratory disease. Ground-glass opacities represent the inflammatory process, and fibrotic abnormalities are a consequence of lung damage in severe long-term disease. Lung CXR is typically normal in patients with COVID-19, and it is not possible to establish whether ILD will arise as a long-COVID complication. Therefore, ILD patients require HRCT to improve diagnostic ability and prognosis in the setting of long COVID [22].

### 7.2. High-Resolution Computed Tomography (HRCT)

HRCT is the preferred imaging modality for the diagnosis, management, and monitoring of post-COVID-19 ILD. HRCT scans identify the location and distribution of lung abnormalities, which can be bilateral, peripheral, or mid- to lower-lobe parenchymal changes. The most common HRCT patterns are ground-glass opacities, indicative of alveolar spaces filled with cells or fluid, alveolar wall thickness, and a reticular pattern, typical of ILD [20]. The extent of HRCT abnormalities correlates closely with symptom severity and TLC reduction, in agreement with National Institute of Health findings [18]. HRCT allows: (1) early fibrosis detection; (2) treatment guidance, including use of antifibrotic drugs; and (3) prognostic assessment via serial imaging or scale evaluation [23].

A potential radiologic sign of HRCT is the atoll sign, or the reversed halo sign. The characteristic image shows a central area of ground-glass opacity surrounded by a ring of denser consolidation. This pattern can be suggestive of organizing pneumonia (OP) and is found in a variety of pulmonary diseases like fungal infections in neutropenic patients, granulomatosis with polyangiitis, and various tumors such as lung adenocarcinoma and lymphoma. Histopathology shows that the central ground-glass opacity corresponds to septal inflammation, while the peripheral ring relates to inflammation of the alveolar ducts or airspaces.

**Figure 3:**
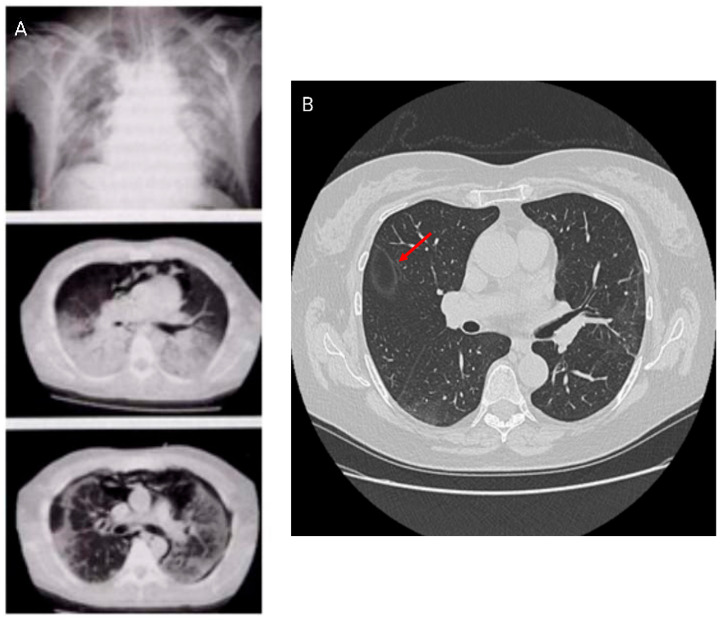
Radiological imaging in COVID-19 infection. HRCT patterns in Acute Respiratory Distress Syndrome (ARDS) (**A**) and in subacute phase with atoll or reversed halo sign ((**B**) arrow).

Further, the crazy paving pattern, characterized by ground-glass opacities with interlobular and intralobular septal thickening, has been observed in HRCT imaging associated with COVID-19. HRCT patterns are non-specific because they can be found in alveolar proteinosis, ARDS, and other interstitial pneumonias [24,25]. Studies have identified different HRCT patterns between early and more advanced disease stages. The latter showed an increase in pleural changes including subpleural lines and effusions. HRCT patterns are shown in Figure 3.

Several HRCT patterns have been reported in IPF, but image-based biomarkers are not routinely used in clinical practice. HRCT scan results provide prognostic information on progression-free survival and mortality in IPF patients [26].

## 8. Interstitial Lung Abnormalities (ILA)

**Figure 4:**
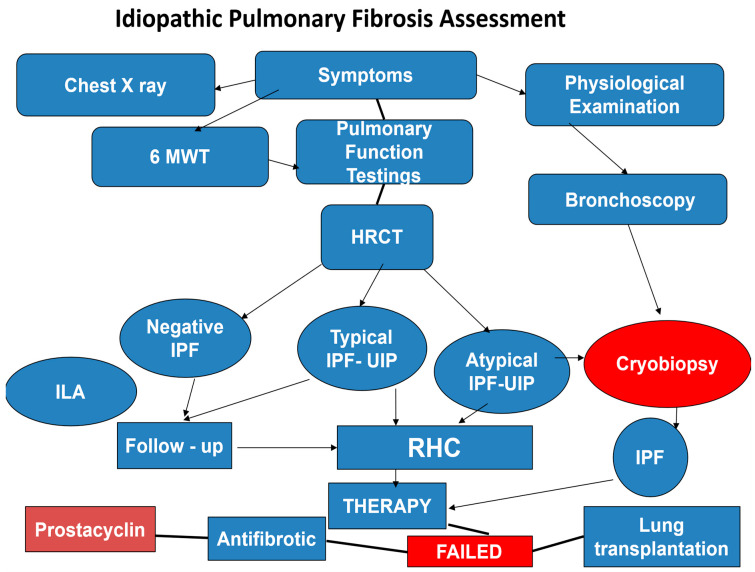
Flow chart summarizing idiopathic pulmonary fibrosis clinical assessment.

ILA are detected by HRCT in the lung parenchyma of approximately 5% of patients without ILD [12]. HRCT features include traction bronchiectasis, ground-glass opacity, reticulation, honeycombing, and non-emphysematous cysts. ILA may evolve into progressive disease within about 2 to 5 years after COVID-19. Interestingly, ILA may precede IPF and other progressive pulmonary fibrotic diseases such as connective tissue disease-associated ILD (CTD-ILD), nonspecific interstitial pneumonia (NSIP), and fibrotic hypersensitivity pneumonia (HP). These subgroups may have ILA-like HRCT findings. The most common radiological finding in ILA is typically usual interstitial pneumonia (UIP). Current studies show that extensive traction bronchiectasis is more frequently found in ILA, compared to other ILD or IPF. Traction bronchiectasis is the most frequent cause of ILA progression and carries a worse 5-year prognosis. Histopathology is difficult to obtain in ILA. Investigations performed multiple biopsies with HRCT and concluded that the UIP pattern was observed in some ILA cases. The most common ILA genetic risk was the rs35705950 gene variant 5B (MUC5B). This gene is a promoter of polymorphisms closely related to familial pulmonary fibrosis sometimes observed in cases of IPF/UIP [12]. The clinical assessment and management of IPF are summarized in the flow chart (Figure 4).

## 9. Cryobiopsy in ILD

Transbronchial lung cryobiopsy (TBLC) has been utilized as a new biopsy approach in ILD since 2009 [27]. Several studies have shown that lung biopsy findings provide valuable information for both diagnosis and prognosis of ILD and help determine the appropriate treatment course [28]. The histological features help in the following ways:

(i) classifying ILDs and distinguishing between idiopathic and secondary forms: for instance, the presence of additional findings like lymphoid aggregates in an NSIP pattern may indicate a CTD-ILD and not an idiopathic NSIP [29]; similarly the presence of scattered granulomas, centrilobular/bronchiolar centered fibrosis, bridging fibrosis, cicatricial organizing pneumonia, and scattered lymphocytic infiltrates favor UIP secondary to fibrotic hypersensitivity pneumonitis rather than idiopathic UIP of IPF.
(ii) evaluating: (a) the presence/absence and type of fibrosis and inflammation, (b) disease distribution, and (c) architectural pattern(s) (e.g., UIP vs. NSIP).
(iii) selecting the initial treatment: i.e., anti-inflammatory vs. antifibrotic therapy. Anti-inflammatory therapy may lead to disease regression in ILD with predominant inflammatory components like hypersensitivity pneumonitis (HP), pulmonary vasculitis, or ILD associated with connective tissue disease (CTD), while antifibrotic therapy may slow disease progression in ILDs with predominant fibrotic disease like IPF and PPF [30].
(iv) impacting treatment strategy and predicting prognosis: a study conducted by Tomasetti et al. [28] showed that lung biopsy data significantly changed the recommended treatment strategy in 34% of ILD cases. In at least a quarter of cases, the initial clinical-radiological diagnosis was revised and reclassified. In these cases, the initial “wait and see” approach of the medical diagnostic team (MDT) before lung biopsy and the inappropriate use of corticosteroids in fibrotic ILDs were reduced, while the recommendation for antifibrotic therapy or immunomodulation was increased. They also found that prognosis of the cases reclassified by the MDT after biopsy significantly differed from that of the initial MDT working diagnosis. The cases classified as non-IPF by a clinician and radiologist and then reclassified as IPF after biopsy had significantly worse survival compared to non-IPF confirmed cases, while cases initially classified as IPF and then reclassified as non-IPF after lung biopsy had a better prognosis.
(v) increasing diagnostic confidence: In the study by Hetzel et al. [31] on the diagnostic confidence of TBLC in ILD, there was a significant increase in the percentage of cases with a diagnostic likelihood above 70%. Diagnostic likelihood rose from 50.0% with clinical-radiological discussion alone to 81.2% after TBLC. There was a significant increase in the percentage of cases with a definitive diagnosis (likelihood ≥ 90%). Certainty increased from 11.7% after clinic-radiological discussion to 53.9% with the integration of TBLC data. This study found that adding TBLC to the clinical assessment increased diagnostic confidence to a level that likely influenced clinical management.

## 10. Cryobiopsy in Post-COVID-19 ILD

As is the case with other ILDs, lung biopsy in post-COVID-19 ILD can help define histological patterns, assess pulmonary fibrosis presence and severity, and help with better understanding of the pathogenic mechanisms, thereby improving consistent, personalized management. Ravaglia et al. [32] evaluated biopsies of patients with clinical and radiologic evidence of residual disease in the post-COVID-19 recovery phase and found three different clusters of histological patterns in the biopsy.

**Figure 5:**
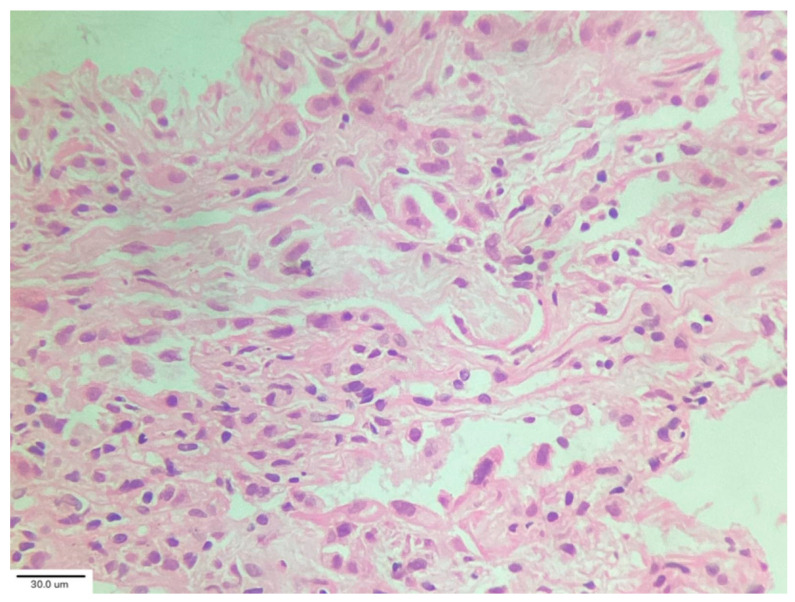
Hyperplastic alveolar epithelial type II cells (AECII). Magnification: H&E ×100.

− Cluster 1 (pre-existing “chronic fibrosing”) was characterized by post-infection progression of pre-existing interstitial pneumonia.
− Cluster 2 (“acute/subacute injury”) was characterized by a great variety of lung injury patterns, including diffuse alveolar damage, organizing pneumonia, and fibrosing nonspecific interstitial pneumonia. The histopathology included diffuse thickening of the alveolar interstitium by myofibroblast proliferation and dense fibrosis. Remnant alveolar spaces showed type II pneumocyte hyperplasia (Figure 5), which expressed phosphorylated signal transducer and activator of transcription 3 (pSTAT3) and Ki67. Nodular lymphocytic infiltrates were noted in the perivascular and interstitial spaces.
− Cluster 3 (“vascular changes”) was characterized by diffuse vascular increase, dilatation of the lumen of the alveolar capillaries and venules, and hyperplasia of capillaries within an otherwise normal or minimally abnormal parenchyma. Endothelial cells of the interstitial capillaries and venules showed strong and diffuse expression of indoleamine 2, 3-dioxygenase (IDO), programmed death-ligand 1 (PDL1), and pSTAT3. This expression profile was not observed in biopsies of healthy subjects or in patients with diffuse parenchymal lung diseases used as uninfected control specimens in this study. They concluded that post-COVID-19 lung disease includes phenotypically different processes warranting different treatment protocols [32].

In the TLBC study by Guedes Baldi et al. [33] that included post-COVID-19 patients with persistent symptoms and ILAs on HRCT, the predominant finding was bronchiolocentric interstitial pneumonia with architectural distortion of the bronchial smooth muscle layer and peribronchial remodeling with extracellular matrix deposition in the alveolar interstitium, demonstrating a fibrotic phenotype.

**Figure 6:**
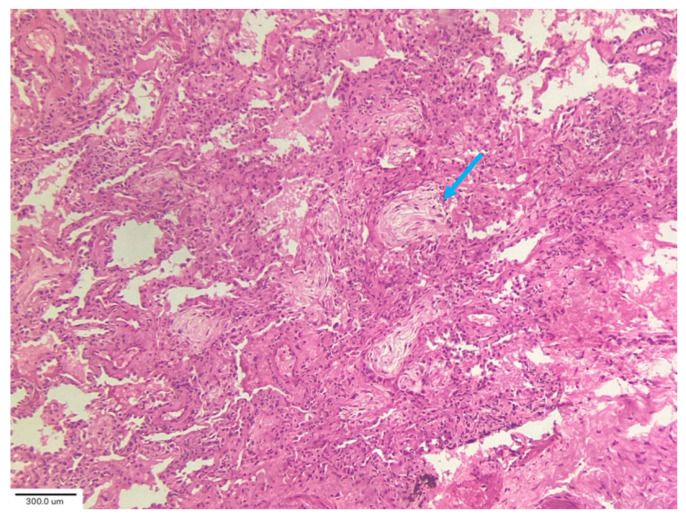
Organizing pneumonia with Masson bodies (arrow) and varying degrees of lymphoplasmacytic interstitial infiltrate. Magnification: H&E ×40.

In their analysis of cryobiopsies in post-COVID-19 patients, Culebras et al. [34] described the following findings: OP with Masson bodies (Figure 6), varying degrees of lymphoplasmacytic interstitial infiltrates and interstitial giant cells, and patchy collagenous interstitial/alveolar scars in the absence of fibroblastic foci. The fibrosis was confined in shape and distribution to “old OP.” No clear irreversible fibrosis or any classic pattern (UIP or NSIP) or smoking-related interstitial fibrosis, was observed. There were no hyaline membranes, fibroblastic enlargement of the interstitium, or thrombi observed in these biopsies. The most common injury pattern encountered on histologic analysis of postmortem and explant biopsies after COVID-19 is diffuse alveolar damage (DAD), the histologic correlate of ARDS. ARDS, both of infectious and non-infectious origin, may progress to the development of significant and irreversible pulmonary fibrosis [35].

**Figure 7:**
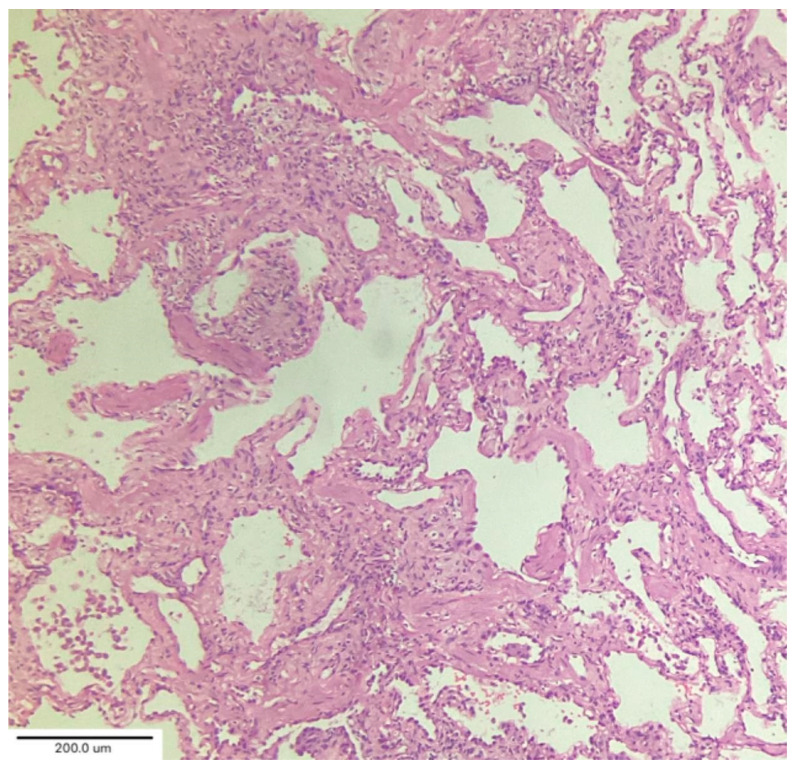
Diffuse interstitial fibrosis with nonspecific interstitial pneumonia pattern. Magnification: H&E ×100.

A case series by Aesif et al. [35] described the presence of diffuse interstitial fibrosis vaguely resembling an NSIP pattern (Figure 7) and areas of microscopic honeycombing in patients after a 4-month follow-up period. Fibroblastic foci were not observed in the biopsies. Incorporating biopsy findings into the post-COVID-19 multidisciplinary evaluation can help to identify key patient subgroups, understand the pathogenic mechanisms of ILD progression, and create better patient-specific treatment protocols that improve clinical outcomes. [36,37]

**Figure 8:**
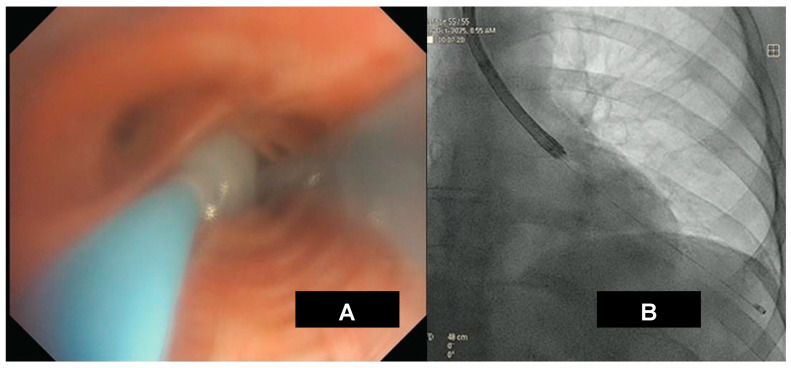
Endobronchial cryobiopsy procedure (**A**) supported by radiological imaging (**B**) of the right inferior lobe. *Courtesy of Pallav L. Shah*.

TBLC procedure is reported in Figure 8.

## 11. Treatment of Post-COVID-19 ILD

Currently, treatment of post-COVID-19 ILD is not yet defined by guidelines or clinical trials. Medical treatment includes corticosteroids, antifibrotic drugs, supplemental oxygen, pulmonary physiotherapy rehabilitation, smoking cessation, and vaccination. Notably, treatment for post-COVID-19 ILD focuses on symptom and inflammation management. Patients with pre-existing ILD require ongoing therapy with reassessment and adjustment, although longer-term prognosis may vary [3]. Newer cases of post-COVID-19 ILD should be considered for antifibrotic therapy such as nintedanib, whose efficacy has been proven pulmonary fibrosis trials [34]. Monitoring is essential, as COVID-19 can worsen ILD, and prognosis may vary depending on the specific ILD type, as reported by Fukihara et al. [38].

Corticosteroid therapy may be initiated empirically, with typical dosing of methylprednisolone (0.5–1.0 mg/kg/day) for 4 weeks, obtaining a good clinical and functional response rate [39]. Steroids treatment reduces the inflammatory process, thereby improving clinical symptoms and decreasing the extension of ground-glass opacity on lung HRCT imaging. Antibiotics like azithromycin or clarithromycin can be used in post-COVID-19 ILD acute exacerbations.

Supplemental oxygen should be carefully titrated, monitoring for potential hypercapnia. Both invasive mechanical ventilation and non-invasive ventilation strategies have defined roles in management and are selected based on disease severity and clinical trajectory. ILD can be exacerbated by excessive airway pressures from inappropriate ventilator settings. Prone positioning has been shown to improve oxygenation by optimizing ventilation–perfusion matching and redistributing pulmonary edema, thereby reducing inspiratory pressure requirements [40].

Antifibrotic drugs such as pirfenidone and nintedanib can be combined with corticosteroids to accelerate recovery. These drugs have become key elements for reducing the rate of progression of post-COVID-19 ILD, including IPF. Recently, nerandomilast, a phosphodiesterase 4 inhibitor, has been recommended for IPF treatment in adult patients (>40 yrs) and was approved by the Food and Drug Administration (October 2025) for IPF. Two randomized double-blind placebo controlled clinical trials (FIBRONEER-IPF 1 and 2) showed that nerandomilast (18 mg twice daily) slowed the decline of FVC (mL) after 52 weeks of therapy. Notably, FVC was greater than or equal to 45% predicted, and DLCO corrected for hemoglobin was greater than or equal to 25% predicted. Secondary end points were time to first acute IPF exacerbation and first respiratory hospitalization or death. The study (up to 109 weeks) found that there was not a significant difference in secondary end points between patients taking 9 mg or 18 mg [41,42,43]. Nerandomilast can be used alone or in combination with pirfenidone in post-COVID-19 ILD. Additional clinical trials are needed to confirm the efficacy of this treatment.

While COVID-19 vaccination remains strongly recommended for all IPF patients and has been thought to play an important role in preventing post-COVID-19 ILD and long COVID, there are case reports of patients developing ILD after receiving the COVID-19 vaccination [44]. In this context, Sgalla et al. [45] suggest that further evidence is needed to clarify whether a specific IPF phenotype might be at higher risk of acute exacerbation following vaccine administration.

## 12. Prognosis of Post-COVID-19 ILD

The prognosis of post-COVID-19 ILD remains uncertain. Several studies report that most survivors experience gradual improvement or stability, whereas other patients may have progressive lung function decline attributable to pulmonary fibrosis development. ILD prognosis varies depending on the specific ILD subtype and frequency of acute exacerbations. Aggressive ILD subtypes such as IPF have a poorer prognosis. Early diagnosis and treatment, including antifibrotic drugs, may reduce disease progression and improve prognosis and life expectancy. Therefore, it remains uncertain whether post-COVID-19 ILD will improve over time, persist and be permanent, or lead to progressive pulmonary fibrosis [46,47].

## 13. Conclusions

Post-COVID-19 interstitial lung disease is a persistent lung condition following COVID-19 infection characterized by long-term respiratory symptoms, radiological findings and abnormalities, and reduced lung function. Risk factors include severe acute illness, advanced age, male sex, and smoking. Current evidence suggests that fibrotic injury is a consequence of the initial infection and could be influenced by persistent inflammation and dysregulated tissue repair. Prevention and treatment guidelines are lacking. Ongoing research explores treatment options such as corticosteroids, new antifibrotic drugs, and regenerative medicine.

Post-COVID-19 ILD represents a significant long-term complication requiring continued research to better understand the long-term complications and management. As research progresses, more effective diagnostic and therapeutic strategies are expected to emerge.

## Data Availability

No new data were created or analyzed in this study. Date sharing is not applicable to this article.

## References

[1] Alrajhi N.N. (2023). Post-COVID-19 pulmonary fibrosis: An ongoing concern. Ann. Thorac. Med..

[2] Saha P., Talwar P. (2024). Idiopathic pulmonary fibrosis (IPF): Disease pathophysiology, targets, and potential therapeutic interventions. Mol. Cell. Biochem..

[3] García-Hidalgo M.C., González J., Benítez I.D., Carmona P., Santisteve S., Moncusí-Moix A., Gort-Paniello C., Rodríguez-Jara F., Molinero M., Perez-Pons M. (2022). Proteomic profiling of lung diffusion impairment in the recovery stage of SARS-CoV-2-induced ARDS. Clin. Transl. Med..

[4] McDonald L.T. (2021). Healing after COVID-19: Are survivors at risk for pulmonary fibrosis?. Am. J. Physiol. Lung Cell. Mol. Physiol..

[5] Puppo F., Carbone R.G. (2024). Interstitial lung disease epidemiology in the past three decades: A narrative review. J. Clin. Med..

[6] Raghu G., Remy-Jardin M., Richeldi L., Thomson C.C., Inoue Y., Johkoh T., Kreuter M., Lynch D.A., Maher T.M., Martinez F.J. (2022). Idiopathic Pulmonary Fibrosis (an Update) and Progressive Pulmonary Fibrosis in Adults: An Official ATS/ERS/JRS/ALAT Clinical Practice Guideline. Am. J. Respir. Crit. Care Med..

[7] Wijsenbeek M., Cottin V. (2020). Spectrum of fibrotic lung diseases. N. Engl. J. Med..

[8] King T.E., Bradford W.Z., Castro-Bernardini S., Fagan E.A., Glaspole I., Glassberg M.K., Gorina E., Hopkins P.M., Kardatzke D., Lancaster L. (2014). A phase 3 trial of pirfenidone in patients with idiopathic pulmonary fibrosis. N. Engl. J. Med..

[9] Flaherty K.R., Wells A.U., Cottin V., Devaraj A., Walsh S.L.F., Inoue Y., Richeldi L., Kolb M., Tetzlaff K., Stowasser S. (2019). Nintedanib in progressive fibrosing interstitial lung diseases. N. Engl. J. Med..

[10] Jegal Y., Kim D.S., Shim T.S., Lim C.M., Do Lee S., Koh Y., Kim W.S., Kim W.D., Lee J.S., Travis W.D. (2005). Physiology is a stronger predictor of survival than pathology in fibrotic interstitial pneumonia. Am. J. Respir. Crit. Care Med..

[11] Raghu G., Remy-Jardin M., Myers J.L., Richeldi L., Ryerson C.J., Lederer D.J., Behr J., Cottin V., Danoff S.K., Morell F. (2018). Diagnosis of idiopathic pulmonary fibrosis. An Official ATS/ERS/JRS/ALAT Clinical Practice Guideline. Am. J. Respir. Crit. Care Med..

[12] Carbone R.G., Puppo F., Russell A.M., Carbone R.G., Puppo F., Levine D.J. (2025). Interstitial lung disease and pulmonary hypertension in advanced lung disease. Pulmonary Hypertension and Lung Transplantation.

[13] Maher T.M., Bendstrup E., Kreuter M. (2021). Global incidence and prevalence of idiopathic pulmonary fibrosis. Respir. Res..

[14] Kaunisto J., Salomaa E.R., Hodgson U., Kaarteenaho R., Kankaanranta H., Koli K., Vahlberg T., Myllärniemi M. (2019). Demographic and survival of patients with idiopathic pulmonary fibrosis in the Finnish IPF registry. ERJ Open Res..

[15] Cottin V., Spagnolo P., Bonniaud P., Nolin M., Dalon F., Kirchgässler K.U., Kamath T.V., Van Ganse E., Belhassen M. (2021). Mortality and respiratory-related hospitalizations in idiopathic pulmonary fibrosis not treated with antifibrotics. Front. Med..

[16] Zhang L., Lu Q., Chang C. (2020). Epigenetics in Health and Disease. Adv. Exp. Med. Biol..

[17] Farsetti A., Illi B., Gaetano C. (2023). How epigenetics impacts on human diseases. Eur. J. Intern. Med..

[18] Shamsi M.B., Firoz A.S., Imam S.N., Alzaman N., Samman M.A. (2017). Epigenetics of human diseases and scope in future therapeutics. J. Taibah Univ. Med. Sci..

[19] Martínez-Besteiro E., Molina-Molina M., Gaeta A.M., Aburto M., Casanova Á., Rigual Bobillo J., Orozco S., Pérez Rojo R., Godoy R., López-Muñiz Ballesteros B. (2023). Impact of COVID-19 infection on patients with preexisting interstitial lung disease: A Spanish Multicentre Study. Arch. Bronconeumol..

[20] Vreeman E.C.A., Pillay J., Burgess J.K. (2025). Post-COVID pulmonary sequelae: Mechanisms and potential targets to reduce persistent fibrosis. Pharmacol. Ther..

[21] Ielapi N., Andreucci M., Bracale U.M., Costa D., Bevacqua E., Francica G., Serraino G.F., Serra R. (2020). Cardiovascular disease as a biomarker for an increased risk of COVID-19 infection and related poor prognosis. Biomark. Med..

[22] Bazdar S., Kwee A.K., Houweling L., de Wit-van Wijck Y., Mohamed Hoesein F.A., Downward G.S., Nossent E.J., Maitland-van der Zee A.H., P4O2 Consortium (2023). A systematic review of chest imaging findings in long COVID patients. J. Pers. Med..

[23] Rahman M.T., Nahar N.U., Ibrahim M., Islam I., Bhowmik B., Shirin M., Khan M.M.R., Akkas N., Molla M.M.A. (2023). Early prediction and HRCT evaluation of post COVID-19 related lung fibrosis. Microbiol. Insights.

[24] Bernheim A., Mei X., Huang M., Yang Y., Fayad Z.A., Zhang N., Diao K., Lin B., Zhu X., Li K. (2020). Chest CT findings in coronavirus disease-19 (COVID-19): Relationship to duration of infection. Radiology.

[25] Zhou S., Wang Y., Zhu T., Xia L. (2020). CT features of coronavirus disease 2019 (COVID-19) pneumonia in 62 patients in Wuhan, China. AJR Am. J. Roentgenol..

[26] Thillai M., Oldham J.M., Ruggiero A., Kanavati F., McLellan T., Saini G., Johnson S.R., Ble F.X., Azim A., Ostridge K. (2024). Deep Learning-based segmentation of computed tomography scans predicts disease progression and mortality in idiopathic pulmonary fibrosis. Am. J. Respir. Crit. Care Med..

[27] Korevaar D.A., Colella S., Fally M., Camuset J., Colby T.V., Hagmeyer L., Hetzel J., Maldonado F., Morais A., Ravaglia C. (2022). European Respiratory Society guidelines on transbronchial lung cryobiopsy in the diagnosis of interstitial lung disease. Eur. Respir. J..

[28] Tomassetti S., Ravaglia C., Puglisi S., Ryu J.H., Colby T.V., Cavazza A., Wells A.U., Pavone M., Vancheri C., Lavorini F. (2022). Impact of lung biopsy information on treatment strategy of patients with interstitial lung diseases. Ann. Am. Thorac. Soc..

[29] Ryerson C.J., Adegunsoye A., Piciucchi S., Hariri L.P., Khor Y.H., Wijsenbeek M.S., Wells A.U., Sharma A., Cooper W.A., Antoniou K. (2025). Update of the International Multidisciplinary Classification of the interstitial pneumonias: An ERS/ATS Statement. Eur. Respir. J..

[30] Behr J., Salisbury M.L., Walsh S.L.F., Podolanczuk A.J., Hariri L.P., Hunninghake G.M., Kolb M., Ryerson C.J., Cottin V., Beasley M.B. (2024). The role of inflammation and fibrosis in interstitial lung disease treatment decisions. Am. J. Respir. Crit. Care Med..

[31] Hetzel J., Wells A.U., Costabel U., Colby T.V., Walsh S.L.F., Verschakelen J., Cavazza A., Tomassetti S., Ravaglia C., Böckeler M. (2020). Transbronchial cryobiopsy increases diagnostic confidence in interstitial lung disease: A prospective multicentre trial. Eur. Respir. J..

[32] Ravaglia C., Doglioni C., Chilosi M., Piciucchi S., Dubini A., Rossi G., Pedica F., Puglisi S., Donati L., Tomassetti S. (2022). Clinical, radiological and pathological findings in patients with persistent lung disease following SARS-CoV-2 infection. Eur. Respir. J..

[33] Baldi B.G., Fabro A.T., Franco A.C., Machado M.H.C., Prudente R.A., Franco E.T., Marrone S.R., Vale S.A.D., Cezare T.J., Moraes M.P.T. (2022). Clinical, radiological, and transbronchial biopsy findings in patients with long COVID-19: A case series. J. Bras. Pneumol..

[34] Culebras M., Loor K., Sansano I., Persiva Ó., Clofent D., Polverino E., Felipe A., Osorio J., Muñoz X., Álvarez A. (2021). Histological findings in transbronchial cryobiopsies obtained from patients after COVID-19. Chest.

[35] Aesif S.W., Bribriesco A.C., Yadav R., Nugent S.L., Zubkus D., Tan C.D., Mehta A.C., Mukhopadhyay S. (2021). Pulmonary Pathology of COVID-19 following 8 weeks to 4 months of severe disease: A report of three cases, including one with bilateral lung transplantation. Am. J. Clin. Pathol..

[36] Shyam T., Atuaka C., Venigalla M., Sinokrot O. (2025). Scars from the pandemic: Understanding post-COVID-19 interstitial lung disease. Breathe.

[37] Wild J.M., Porter J.C., Molyneaux P.L., George P.M., Stewart I., Allen R.J., Aul R., Baillie J.K., Barratt S.L., Beirne P. (2021). Understanding the burden of interstitial lung disease post-COVID-19: The UK Interstitial Lung Disease-Long COVID Study (UKILD-Long COVID). BMJ Open Respir. Res..

[38] Fukihara J., Kondoh Y. (2023). COVID-19 and interstitial lung diseases: A multifaceted look at the relationship between the two diseases. Respir. Investig..

[39] Yüksel A., Karadoğan D., Hürsoy N., Telatar T.G., Köse Kabil N., Marım F., Kaya İ., Er A.B., Erçelik M., Polat Yuluğ D. (2022). Post-COVID-19 Interstitial Lung Disease: A new entity, what do we deal with?. Eur. Respir. J..

[40] Sauleda J., Nuňez B., Sala E., Soriano J.B. (2018). Idiopathic pulmonary fibrosis: Epidemiology, natural history, phenotypes. Med. Sci..

[41] Maher T.M., Assassi S., Azuma A., Cottin V., Hoffman-Vold A.M., Kreuter M., Oldham J.M., Richeldi L., Valenzuela C., Wijsenbeek M.S. (2025). Nerandomilast in patients with progressive pulmonary fibrosis. N. Engl. J. Med..

[42] Richeldi L., Azuma A., Cottin V., Kreuter M., Maher T.M., Martinez F.J., Oldham J.M., Valenzuela C., Clerisme-Beaty E., Gordat M. (2025). Nerandumilast in patients with idiopathic pulmoanry fibrosis. N. Engl. J. Med..

[43] George P.M., Wells A.U., Jenkins R.G. (2020). Pulmonary fibrosis and COVID-19: The potential role for antifibrotic therapy. Lancet Respir. Med..

[44] Forchette L.T., Palma L., Sanchez C., Gibons R.M., Stephenson-Moe C.A., Behers B.J. (2025). Cardiopulmonary effects of COVID-19 vaccination: A comprehensive narrative review. Vaccines.

[45] Sgalla G., Magrì T., Lerede M., Comes A., Richeldi L. (2022). COVID-19 Vaccine in patients with exacerbation of idiopathic pulmonary fibrosis. Am. J. Respir. Crit. Care Med..

[46] Hajari Case A. (2023). Post COVID-19 interstitial lung disease. *Pulmonaryfibrosis.org*. Post COVID-19 Interstitial Lung Disease. https://www.pulmonaryfibrosis.org/docs/default-source/programs/educational-materials/fact-sheets-english/pf-series---covid7e0065905fc24090a1ecd15f23ef2318.pdf?sfvrsn=f0c22518_2.

[47] Kewalramani N., Heenan K.M., McKeegan D., Chaudhuri N. (2023). Post-COVID interstitial lung disease-the tip of the iceberg. Immunol. Allergy Clin. N. Am..

